# The impact of knowledge management on the quality of services in nursing homes

**DOI:** 10.3389/fpsyg.2022.1106014

**Published:** 2023-01-19

**Authors:** Anamarija Kejžar, Vlado Dimovski, Simon Colnar

**Affiliations:** ^1^Faculty of Social Work, University of Ljubljana, Ljubljana, Slovenia; ^2^School of Economics and Business, University of Ljubljana, Ljubljana, Slovenia

**Keywords:** teamwork, quality management, knowledge management, tacit knowledge, E-Qalin

## Abstract

**Introduction:**

Current management strategies in nursing homes (NH) aim to ensure effective knowledge management (KM) in order to provide both best possible services to residents, and care for staff in NH. Teamwork in NH is essential for effective delivery of the highest quality of services. As a result, NH are increasingly adopting KM activities to enable knowledge creation, storage, transfer, and implementation in an environment facing many challenges such as lack of staff, increasing demands, and expectations of residents.

**Methods:**

In our quantitative study, we examined how two Slovenian state NH that adopted the E-Qalin quality management model (European quality-improving learning model) adapted their KM, and what impact their quality management system and KM activities actually have on the quality of services. Furthermore, we examined how two Slovenian private NH that have not adopted a certified quality management program (like ISO or E-Qalin) tackle the issue of quality of services from the KM perspective. The sample consisted of 80 nursing professionals. In every NH that is part of this study, teamwork is essential and every individual that was involved in our survey is part of a team. In our study, we analyzed relationships between individual variables using linear regression.

**Results:**

We found a significant and positive relationship between knowledge creation, transfer, and implementation in NH with and without the E-Qalin certificate. We found a significant and positive relationship between knowledge storage and the quality of services only in NH without a E-Qualin certificate. It seems that when connecting multidisciplinary fields such as NH and KM, there is still a lack of awareness and knowledge on the topic of KM, which might be one of the reasons for some bias in the answers provided by respondents. We also found different teamwork approaches in NH with and without a E-Qalin certificate. Our research results therefore emphasize the need to gain additional insight into quality management and KM in the environment of NH.

**Conclusion:**

Teamwork based on knowledge storage, transfer, the implementation of existing knowledge, and creation of new knowledge are essential for well-trained professionals and, as a result, contribute to continuous improvement in service quality. Implementation of KM is well received by NH, and enables them to better meet the needs and expectations of residents. More importantly, nursing staff also share and pass on tacit knowledge through teamwork. Finally, all of the NH in our study that implemented quality and KM activities noted an improvement in the quality of services that are offered to residents in practice. Our results indicate that the topic of KM in NH is interesting, and it has a positive impact on the quality of services in practice. However, the problem of awareness and knowledge on the topic of KM in the environment of NH still exists, highlighting the need for further research, additional insight, and dissemination of knowledge to every interested stakeholder functioning in the field of NH. The results of the study make an important contribution to the research of KM in NH, focusing on the transfer of tacit knowledge.

## 1. Introduction

The term “nursing home” is an oxymoron, as Tirrito (2003) stated – nursing homes (NH) are medical facilities that infrequently resemble homes even if the furnishings are home-like. The routines of care, the concern for regulatory procedures, and the lack of individualization are institutional and not home-like (Tirrito, 2003). Therefore, on the one hand, NH want to maintain the highest possible standards of care, but at the same time they aim to change the very philosophy of long-term care by involving users (residents) in the planning of the care process. Long-term care places users at the center of care as co-creators of quality. As a starting point for designing new solutions and creating new knowledge, we can use the concept of quality of life, where the user himself is the most important in planning his/her care. Everyone determines the quality of life and aging, based on own ideas, expectations, goals, and abilities. Therefore, the quality of care is based on the personalization of care, which is co-created by both care providers and users themselves. We cannot imagine the quality of services in NH as long as only employees (as experts) make decisions, and that users are not involved in planning their own care.

In Slovenia, quality management according to the E-Qalin certificate is the most widespread, and this article compares it with the quality management of the DEOS d.o.o. company (the largest private company of nine NH in Slovenia). Many older people living in NH have high levels of dependency, cognitive impairment, and multimorbidity, with daily unexpected events that can threaten the health or even life of the resident, and so knowledge transfer, the creation of new knowledge, and innovations tailored to the person that are coherent, consistent with protocols, and focused on the concept of person-centered care, are essential from the perspective of the quality of life of a person in a NH. In addition, healthcare personnel working in NH are under strong pressure to provide good care to the residents (Rinnan et al., 2018; André et al., 2021). In contrast, it is difficult to recruit and retain qualified workers (Tourangeau et al., 2017; Grødal et al., 2019; André et al., 2021) in this time of a lack of nursing staff worldwide. In Slovenia, 59 public institutions and 43 concession (102 NH) providers offer long-term care (institutional care, day care, and home care) with the capacity to care for 21,150 elderly people (Kejžar et al., 2022). Consequently, the majority of NH are part of a public network, but the number of private care homes holding a concession has been growing over the last decade (Hlebec and Rakar, 2017 in Filipovič Hrast and Rakar, 2021).

In Slovenia, there is no comparative or evaluative scale for the quality of services in NH. The NH management decides independently whether to be certified in order to improve the quality of the NH services and to increase its reputation in the local community. While E-Qalin quality management is done according to the principle of multidisciplinary teams together with users (residents of NH), the quality system of the private company DEOS (which owns nine NH) performs its own quality assessment system. This leads to knowledge transfer inside each home, as well as in all DEOS system facilities in the form of monodisciplinary teams, where residents are not members of the team. We need a new paradigm of quality services in long-term care that places the user at the center of planning person-centered care, that is based on teamwork, which enables the transfer of individual and organizational knowledge in NH, and is consistent with the paradigm of long-term care that links and envisions the coordinated functioning of health and social care.

## 2. Theoretical background and hypotheses development

This study contributes to establishing a connection between quality management and KM in NH for achieving the highest possible quality of services for residents through KM. Despite the entry into the NH market of many new facilities, demand outstrips supply. Many NH operate at 100% capacity, and NH generally do not face much competitive pressure. Quality issues persist, and health and safety standards continue to be developed and implemented (Castle and Ferguson, 2010). The article points out the importance of quality management and teamwork, which contribute to KM in NH, i.e., knowledge creation, storage, transfer, and implementation.

In line with the new paradigm of long-term care, some NH also involve users in regular team meetings. We must be aware that in the process of continuous quality improvement we learn and change based on the wishes, expectations, and needs of users and the aging society, and in this process we create new knowledge – together with users. The user in long-term care is no longer just a passive recipient of services, but has a significant influence on the way and form in which long-term care services are delivered. A wider view in long-term care pushes the boundaries of participation in innovations in long-term care with users, carers, and community groups in the ongoing changing process of teamwork (Gabriel et al., 2017; Senior, 2019). Teams are viewed as knowledge-integrating mechanisms, and it is through teamwork that individuals’ knowledge can be shared and mobilized (Erhardt, 2011; Du et al., 2021). Knowledge can be found in different forms such as codified individual knowledge, written documentation, documented procedures, and processes of especially tacit knowledge from individuals and networks of individuals (Donate and Pablo, 2015).

## 3. Knowledge management in nursing homes and its influence on quality of services

**Figure 1:**
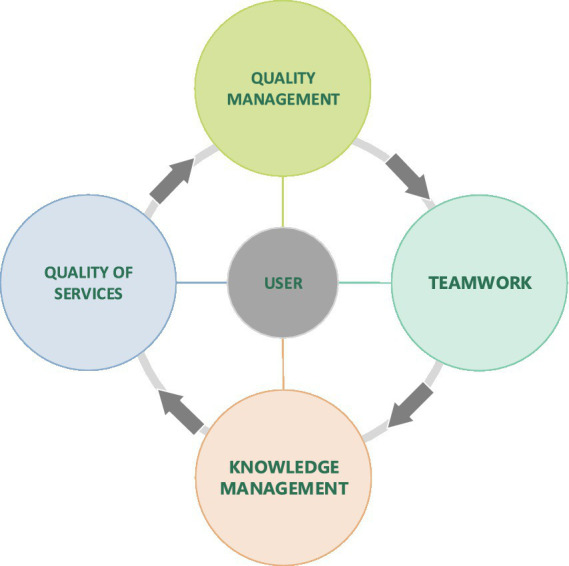
User oriented quality management in nursing homes model shows the interdependence of teamwork, quality management, knowledge management with the quality of user-oriented services. For quality care services in homes it is important to know the habits of the user, his wishes and the implementation of person-centered care.

Knowledge management is the process of capturing, developing, sharing, and effectively using organizational knowledge. Knowledge management is a set of principles, tools, and practices that enable people to create knowledge and help them share, translate, and use it to create greater value and improve efficiency (Girard and Girard, 2015). Utilizing knowledge in an effective and consistent manner is an opportunity for organizations to gain a competitive advantage. Knowledge management activities enable that all of the knowledge within the organization becomes available to everyone (Bolisani and Bratianu, 2017; Rabeea et al., 2019). Moreover, knowledge has become the core competency and a primary source of competitive advantage and is crucial for value creation for all organizations (Li et al., 2018). The existing research supports the claim that KM is becoming an increasingly integral capability for organizations both in the public and private sectors (Al Ahbabi et al., 2018; Gaviria-Marin et al., 2019). However, most research exploring the concept of KM within public sector organizations focuses on health, education, and government intervention (McEvoy et al., 2019), partially neglecting the field of long-term care. In a similar vein, the research of Butler et al. (2008) proposed that KM within the public sector environment could lead to higher efficiency in the offered services (Figure 1).

In our case that focuses on the context of NH, understanding and emphasizing the importance of KM in the environment of the public and private sectors will contribute toward more effectively using KM activities in teamwork, which will then result in a high quality of services in NH. Nowadays, the functioning of public sector organizations can be labeled as extremely complex, as it is a combination of hierarchy and bureaucracy accompanied by service-oriented processes. Therefore, we can argue that the public sector context is one of the most challenging organizational environments in today’s knowledge intensive economy (McEvoy et al., 2019). As such, public sector organizations have to keep up with global trends and meet public demand. This includes the necessity of long-term care in times of an aging population, which further stresses the importance of appropriate and effective KM strategies in NH. On the basis of such characteristics, NH require tailored KM strategies (McEvoy et al., 2019), that take the user’s opinion into account when planning care services – that is, not for the user, but together with him/her.

Public sector organizations are more and more exposed to pressures to improve in terms of productivity and the quality of services that they deliver in practice, which reiterates the necessity to engage in KM activities in order to successfully fulfill their strategic goals (Parker and Bradley, 2000; Abdullah and Date, 2009). By implementing specific elements related to KM activities, such as knowledge transfer, quality of service provision in areas such as healthcare and long-term care, organizations functioning in those sectors have the potential to improve (Gorry, 2008). Given today’s economic challenges, human resources are the most important factor for organizations. This has a significant impact on the quality of service delivery, the existing knowledge of the nursing staff, and the creation of new knowledge. Rising care needs and shrinking workforces are putting pressure on the quality and continuity of long-term care for the elderly. The need to attract and retain a solid workforce is becoming increasingly important. In general, long-term care is a low-wage and low-skill sector where hierarchy, rules, and (cost) efficiency prevail. The underpaid and undervalued work is mainly done by less educated women, and the shortage of nurses interested in working in NH has been critical in recent years and causes the flow of immigrant workers. The proper understanding of the language, organization rules, and normative standards is not self-evident. The important aspect of KM in NH is also the nurse’s intelligent use of all this knowledge that makes her an efficient professional who makes the best out of scarce resources. Her work cannot be standardized nor predetermined (Purkis and Bjornsdottir, 2006) from the person-centered care view.

It has also been suggested that staff behavior is incorporated into tacit routines; they perform tasks that are taken for granted by most people, including themselves (Sandvoll, 2017). Particular care providers played key roles in setting a tone, acting as a role model, mentoring, transforming mistakes into learning opportunities, or sharing key examples (Cammer et al., 2014). New regulations are rarely acknowledged among staff and have little influence on their work. This is in line with the concept of *habitus*, where carers might say “we just do it.” This is an example of aspects of their practice that are tacit or unconscious, and are therefore difficult to explain. In one sense, “tacit practice” might sound like something easily done in everyday care, but the fact is that the term represents a significant body of staff experience and knowledge. However, many of daily routines are based on tacit knowledge among staff because they are unwritten and do not appear in any care plan. The staff just know how the different residents want their routines arranged and “just do it.” Although caring is often understood as the core of nursing, services for older people frequently emphasize visible tasks such as treatment and symptom management, while aspects related to caring seem tacit (Hall and Høy, 2012). It is also troubling that expert skills in the care of older people are often invisible and therefore unrecognized (Phelan and McCormack, 2016).

### 3.1. Knowledge creation in nursing homes and the influence on quality of services

Knowledge creation can be explained as a system of different activities and initiatives that contribute toward generating news ideas or products (Mitchell and Boyle, 2010). In the process of knowledge creation, individuals and teams within an organization and between organizations engage in sharing of tacit and explicit knowledge (Choi and Lee, 2002). Typically, knowledge creation occurs through individual experience from knowledge acquisition, communication and reflection, and organizational knowledge learning (Li et al., 2018). Knowledge creation can be labeled as the engine of innovation as this part of the KM process drives economic and social value. Moreover, knowledge creation enables organizations to create new knowledge, which can be understood as increasing their knowledge assets (Konno and Schillaci, 2021). Knowledge creation can have multiple internal and external organizational sources, and is also an antecedent that allows for the effective use of knowledge. Another characteristic is that it is based on collaboration of professionals from different organizational departments that engage in teamwork. Understanding the implications of knowledge creation is also important for managers in different organizations as knowledge is influential for organizational success, and they need to find ways to improve it, which is possible by encouraging employees to communicate, interact and collaborate, and ask for help (Stojanović-Aleksić et al., 2019), where teamwork has an essential role. In a similar vein, McEvily and Chakravarthy (2000) and Tiwana (2004) also state that numerous empirical studies argue that knowledge creation has the potential of improving organizational performance and quality of services. With our study of knowledge creation in NH and its influence on the quality of services, we partially fill a gap identified in existing literature that is necessary to enhance the understanding of the knowledge creation process (Stojanović-Aleksić et al., 2019). We believe that knowledge creation as part of the overall KM process has the potential to contribute to improving the overall quality of services that are offered to people in practice in NH.

We provide additional empirical research on the relationship between knowledge creation and the quality of services within the environment of NH:

*H1*: Knowledge creation is positively related to the quality of services in nursing homes.

*H1a*: Knowledge creation is positively related to the quality of services in nursing homes with an E-Qalin certificate.

*H1b*: Knowledge creation is positively related to quality of services in nursing homes without an E-Qalin certificate.

### 3.2. Knowledge storage in nursing homes and its influence on quality of services

Knowledge storage can be described as procedures and systems for storing, retrieving, and managing knowledge (Alegre et al., 2013). These are often information-communication technology-based systems that provide support and enhance the overall organizational knowledge storage and retrieval. Employees have the ability to create and acquire relevant knowledge, however, it is also possible that we as individual sometimes forget what we learn. So, knowledge storage represents an important characteristic of effective KM practices in organizations (Alavi and Leidner, 2001). After the initial phase of KM, knowledge creation, there is the necessity to codify and store knowledge in a knowledge database or similar knowledge-based system so that it is ready for retrieval, application, or reuse by other employees or teams in organizations (Samoilenko and Nahar, 2013). Prior research argues that even if an organization is able to obtain new knowledge, it does not necessarily have the ability to also store knowledge (Argote et al., 1990). Such an insight further highlights the necessity of adequately capturing such knowledge and making it available for the whole organization in the long term (Stein and Zwass, 1995).

Due to the increasing volume of knowledge, the constant changes in knowledge, and related activities and technologies, it is complex to store, update, search, and maintain an adequate knowledge base. As knowledge is dynamic in nature, it is important to also remove knowledge that becomes obsolete and replace it with up-to-date relevant knowledge (Wong and Aspinwall, 2005). It is also important to recognize that employee knowledge is valuable to the NH, as well as residents’ expectations and wishes. At the same time, we need to know what kind of knowledge we need and how we can ensure knowledge transfer between employees, since most knowledge exists within employees, in internalized or tacit forms rather than in an explicit (explicit) form. Such a situation is also present in NH. Moreover, in any environment it becomes important to identify and utilize the best available information-communication technology solutions available for the storage and later retrieval of knowledge (Samoilenko and Nahar, 2013). Especially in the environment of NH, knowledge storage and retrieval challenges remain inadequately explored. Understanding how to enable long-term storage and retrieval of explicit and especially tacit knowledge is integral as it represents an important element of organizational memory (Anand et al., 2021). Nursing homes are a repository of knowledge, experience, examples of good practice, and ideas in long-term care. For high-quality services, it is necessary to establish a KM system that preserves and strengthens knowledge in NH. Appropriate usage of storage and retrieval systems is relevant in maintaining internal organizational knowledge and is positively associated with reducing costs and as a prerequisite of efficient knowledge transfer (Taraszewski, 2017). Moreover, knowledge storage as an important part of the KM process has a significant impact on achieving the desired organizational performance outcomes in organizations (Alavi and Leidner, 2001), which also applies to the functioning of NH.

In our research, we gain additional empirical data on the relationship between knowledge storage and quality of services within the environment of NH:

*H2*: Knowledge storage is positively related to the quality of services in nursing homes.

*H2a*: Knowledge storage is positively related to the quality of services in nursing homes with an E-Qalin certificate.

*H2b*: Knowledge storage is positively related to the quality of services in nursing homes without an E-Qalin certificate.

### 3.3. Knowledge transfer in nursing homes and its influence on quality of services

Knowledge transfer is considered as a fundamental activity of KM, as it facilitates the ability of organizations to transform their knowledge into organizational assets and resources (Dawson, 2001). Kumar and Ganesh (2009) define knowledge transfer as different activities of sharing explicit or tacit knowledge between two parties, where one receives and applies the knowledge that was provided by the other. Knowledge transfer also refers to the movement of knowledge through different channels between individuals and teams (Abou Hashish, 2017; Hassan et al., 2017; Tassabehji et al., 2019; Xuan, 2020) or an organization (Joshi et al., 2007). Knowledge transfer is additionally influenced by both the employee willingness to interact with their colleagues and their openness to learn from their colleagues (Ibidunni et al., 2018). This is very important for the mentoring process, where new nursing staff acquire the necessary skills in teamwork and mentoring.

Existing research posits that knowledge transfer has important consequences for a number of organizational processes and outcomes, namely the sharing of best practices (Szulanski, 1996), organizational learning (Reagans et al., 2005) and organizational performance (Hansen, 1999), which can also be understood as the quality of services in the context of NH. Similarly, Argote and Fahrenkopf (2016) support the claim that knowledge transfer is an important tool for improving the performance of individuals, teams, and organizations as the recipients of new knowledge. Additional existing research provides further support for knowledge transfer due to its part in improving organizational performance (Van den Hooff and De Ridder, 2004), while some studies even consider knowledge transfer as a key factor that contributes toward improving organizational performance (Sheng et al., 2013; Oyemomi et al., 2016). In spite of the significant benefits that knowledge transfer brings to organizational performance, knowledge transfer does not automatically happen in organizations (Argote and Miron-Spektor, 2011). Moreover, the up-to-date literature that examines the impact of knowledge transfer on the quality of different services (Radević et al., 2021) in NH remains scarce.

In the opinion of Zaim et al. (2019), organizations should enhance their efforts to promote knowledge transfer as it enables them to improve their overall performance. Another potential benefit of knowledge transfer is its ability to improve the capacity of organizations to function by influencing and improving their core values (Sheng et al., 2013). Due to the numerous positive effects of knowledge transfer on organizational performance, we further highlight the necessity to also regularly update the organizational knowledge base and pursue toward creating new knowledge in order for any type of an organization to gain or maintain their competitive edge and in the case of NH to have better quality services that are also dependent on knowledge transfer between individuals, teams, and organizational units (Radević et al., 2021).

With our paper, we additionally explore the constructs of knowledge transfer and quality of services in the context of NH:

*H3*: Knowledge transfer is positively related to the quality of services in nursing homes.

*H3a*: Knowledge transfer is positively related to the quality of services in nursing homes with an E-Qalin certificate.

*H3b*: Knowledge transfer is positively related to the quality of services in nursing homes without an E-Qalin certificate.

### 3.4. Knowledge implementation in nursing homes and its influence on quality of services

Knowledge implementation is explained as the application of knowledge that an individual or a team have (Lee et al., 2013). It is considered as an organizational tool to store, retrieve, access, and apply knowledge effectively to reach the strategic purposes and goals of an organization (Gold et al., 2001). To gain additional insight into the topic of knowledge implementation, Obeidat et al. (2016) state that it is defined as a combination of social, technological, and operational aspects. Knowledge is only really important when it is utilized and applied in practice to create added value for any type of an organization. Knowledge implementation in general can also be described as the process of using knowledge for a specific purpose, and this can occur when knowledge is deliberately put into action to support managers, employees, decision makers, and policymakers. Without being applied, knowledge does not hold any real value (Al Ahbabi et al., 2018). Such a state suggests that other capabilities of an organization such as knowledge creation, storage, and transfer can be very vague if that organization does not have the ability to apply and use the knowledge in practice in an effective and efficient manner (Mahmoudsalehi et al., 2012).

**Figure 2:**
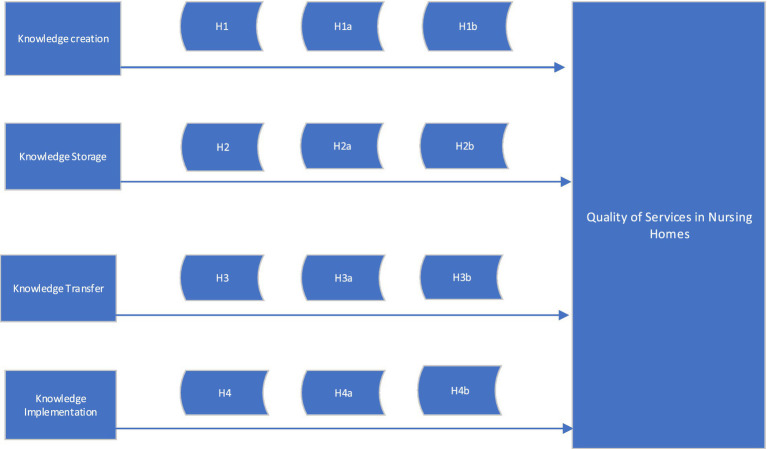
Within the conceptual model of relationships between knowledge creation, storage, transfer and implementation and quality of services in NH, we investigated whether there are differences between new knowledge creation, knowledge transfer, knowledge storage and knowledge implementation in two public nursing homes with a quality certificate E-Qalin and two private DEOS nursing homes with their own quality system.

When knowledge is implemented in practice it also becomes part of the organizational behavior, memory, and processes that are utilized for solving complex problems in the organization, and on a daily basis within the functioning of the organization (Chen et al., 2012). Existing literature additionally empirically supports that knowledge implementation facilitates organizational performance (Mardani et al., 2018). Similarly, Martelo-Landroguez and Cepeda-Carrión (2016) state that knowledge implementation is one of the key organizational processes as it positively influences organizational performance. On an individual level, the positive outcome of knowledge utilization is that it expands one’s expertise and experience and in the long-term enables them to become an expert in their work (Al Ahbabi et al., 2018). Organizations are therefore advised to support employees to use and utilize their knowledge and to rely on an organization’s knowledge repository as a way toward solving existing problems as well as to propose new ways of doing things. This implies the ability to improve the overall quality of services (Chan and Chao, 2008), which is especially relevant in the context of NH (Figure 2). However, it is important to reiterate that knowledge holds little value until it is directly applied in the practice of an organization (Chen and Huang, 2009). Moreover, knowledge implementation is considered as one of the key activities that can help increase individual employee performance in organizations (Bosch-Sijtsema et al., 2009).

Understanding the influence of knowledge implementation on the quality of services in the context of NH requires additional research:

*H4*: Knowledge implementation is positively related to the quality of services in nursing homes.

*H4a*: Knowledge implementation is positively related to the quality of services in nursing homes with an E-Qalin certificate.

*H4b*: Knowledge implementation is positively related to the quality of services in nursing homes without an E-Qalin certificate.

### 3.5. Teamwork in quality management and knowledge management in NH

E-Qalin is a Europe-wide model of quality management in social institutions, the development of which was supported by the European Community. It was created due to the requirements of the development of modern society, social policy, and the social care system. Its creation was conditioned by the requirements of the development of social welfare organizations, their need for quality standards and for a specific model of quality management, which should be recognized nationally and on a European level. Participants were organizations or individuals from Austria, Slovenia, Italy, Germany, Luxembourg, and the Netherlands. In the pilot phase, the model was tested by 29 NH from Austria, Germany, Italy, Luxembourg, and Slovenia, after which an evaluation was carried out and the final version for NH was produced. The quality management system includes the structures, processes, and results of the NH. The quality of all three areas is checked with the help of a self-assessment. The expected effects of quality management according to E-Qalin are mainly: better service quality for users, higher staff motivation and satisfaction, support for management and staff in facilities in providing and improving service quality, national and international comparability of facilities, and certification of a specific activity and brand. The E-Qalin model is currently distributed in the following countries: Austria, Czech Republic, France, Croatia, Italy, Germany, Luxembourg, Slovenia, and the United Kingdom. In NH, after the training of the group leaders and facilitators, the self-evaluation of the criteria is started. According to public data, 21 nursing homes have the certificate E-Qalin so far in Slovenia (Firis, 2022).

Nursing homes managers state that they have noticed an improvement in relationships, communication, and transfer of knowledge in the NH after the implementation of E-Qalin. In the E-Qalin system, there are regular multidisciplinary team meetings involving each of the different professional groups and residents, and there is no longer a problem or issue that concerns only one unit of the home, but a new organizational culture is developed – “*we all work for the benefit of our residents*.” Innovations and implementations that arise on team meetings are stored in information system, where they are available to all staff and for evaluation of changes (Kejžar et al., 2020).

The company DEOS d.o.o. performs the activities of institutional care for the elderly (87,300 – the activity of accommodation facilities for the elderly and disabled), social services in assisted living apartments and home care (DEOS, 2022). DEOS has developed an internal quality management system to monitor and develop quality, which also consists of various criteria. Unlike E-Qalin quality management, the employees of the different units do not meet weekly, but the monodisciplinary teams (the same professionals from each home and without residents) meet regularly and discuss protocols and search for improvements. This means that they can monitor the quality and deviations within their NH in constant comparison with the results and developments in the other seven NH. The structure of team is monodisciplinary with clear protocols and procedures, achieved goals, and results which are monitored monthly. The quality system is managed by quality manager, who also takes care of organizational knowledge in the data base.

## 4. Materials and methods

### 4.1. Participants and procedure

To obtain primary data from our respondents, we utilized an adapted online and in-person questionnaire. Our research was conducted in 2022 on a sample of four NH in Slovenia, of which two (state NH) have the E-Qalin certificate and two (private NH) have their own quality management and consist of 80 employees. To elaborate on some of the most important characteristics of our sample, 76.6% respondents were women, 16.9% respondents were men, while 6.5% respondents did not want to provide an answer regarding their gender. The largest share of respondents (36.4%) belongs to the age cohort from 20 to 29 years. The second largest share of our respondents (28.6%) belongs to the age cohort from 40 to 49 years. The vast majority of our respondents (64.9%) had successfully acquired a high school diploma. Moreover, our respondents had been working in the NH on average for ~8 years (Table 1).

**Table 1:** Sample demographics.

Respondent’s characteristics	Frequency (*N* = 80)
*Gender*	
Men	16.9%
Women	76.6%
No answer	6.5%
*Age*	
Below 20 years	2.6%
20–29 years	36.4%
30–39 years	15.6%
40–49 years	28.6%
50–59 years	11.7%
Above 60 years	5.1%
*Education*	6.5%
Primary school	64.9%
High school	7.8%
Post-secondary Programs	7.8%
First Bologna degree	7.8%
Master of Science	1.3%
PhD	3.9%

We collected data from employees in NH that either have or do not have the E-Qalin certificate by using convenient sampling. Despite the fact that random sampling is often considered the gold standard of research sampling strategies, due to its ability to influence biases and the possibility to evaluate the reliability of the resulting estimates (Tiwari and Chilwal, 2014) researchers are often faced with a trade-off between the desire for randomization and pragmatic considerations when deciding on the sample. As was in the case of our research, random sampling is not always feasible and possible in practice given our constraints in time and resources. Existing research posits that purposive strategies also have the ability to ensure representative samples (Toppl et al., 2004). Moreover, data for all our variables were obtained from individual respondents in a one-time single survey. Therefore, common method bias might potentially have an influence on some of the hypothesized relationship in our research model. To explore the presence of common method bias, we opted to apply Harman’s single factor test (Harman, 1976). The first factor accounted for 59.7% of the overall variance. As this result is slightly above the recommended threshold (50.0%) as supported by existing literature such as Podsakoff et al. (2006), we posit that common method bias might be an issue in our research study.

### 4.2. Measures

To gain insight into individual constructs, we selected measurement scales that are in line with predetermined criteria: (a) often cited in novel research papers that are recently published in relevant scientific journals; (b) they have been utilized in contemporary literature; and (c) are well established and commonly used by prominent authors of our researched topics. In the context of our study, we opted for the five-point Likert scale ranging from 1 (I strongly disagree) to 5 (I completely agree) to gain insight into the respondent’s agreement about the presence of knowledge creation, knowledge storage, knowledge transfer, knowledge implementation, and their influence on the quality of services that are offered in practice by NH.

#### 4.2.1. Knowledge creation

We used the four item scale (*α* = 0.86) that Donate and Pablo (2015) utilized to measure knowledge creation. The questionnaire includes statements such as: “*There is a strong commitment to maintain highly qualified personnel to internally develop or improve services and processes*.”

#### 4.2.2. Knowledge storage

We used the 7-item scale (*α* = 0.90) that Donate and Pablo (2015) opted to use to measure knowledge storage. The questionnaire includes questions such as “*There are databases or repositories that allow employees to use knowledge and experiences that have previously been loaded into the databases*.”

#### 4.2.3. Knowledge transfer

We utilized the 8-item scale (*α* = 0.93) that Donate and Pablo (2015) used to measure knowledge transfer. The questionnaire consists of items such as: “*There are communities of practice and learning groups to share knowledge and experience*.”

#### 4.2.4. Knowledge implementation

We utilized the 4-item scale (*α* = 0.80) from Donate and Pablo (2015) intended to measure knowledge implementation. An example of an item included in the questionnaire is: “*Suggestions from employees, patients, and other important stakeholders are frequently incorporated into services and processes*.”

#### 4.2.5. Quality of services in nursing homes

We used the 20-item scale (*α* = 0.97) that Donate and Pablo (2015) used to measure quality of services. Items in the questionnaire included questions such as: “*Employees/doctors have the customer’s best interest at heart*.”

In our research, we also included several control variables as they can have a significant impact on research conclusions (Bernerth and Aguinis, 2016). Namely, we opted for age, gender, highest level of obtained education, position in organization, years working for this organization, and E-Qalin certificate as our control variables.

## 5. Results

Relationships between individual variables were analyzed using linear regression. Results from Table 2 suggest that respondents on average evaluate knowledge creation (3.80) and quality of services in nursing homes (3.80) the best, which is followed by knowledge storage (3.66). Knowledge transfer (3.47) and knowledge implementation (3.46) on average received a slightly lower evaluation. Correlation coefficients between our measured variables are moderately positive, strongly positive, and very strongly positive with ranges between 0.42 and 0.81 (Akoglu, 2018) and are presented in Table 2.

**Table 2:** Mean values, standard deviations, and coefficient correlations (*n* = 80).

Variable	Mean	SD	1	2	3	4
1. Knowledge creation	3.80	0.84				
2. Knowledge storage	3.66	0.70	0.58**			
3. Knowledge transfer	3.47	0.81	0.50**	0.70**		
4. Knowledge implementation	3.46	0.75	0.51**	0.61**	0.81**	
5. Quality of services	3.80	0.76	0.55**	0.42**	0.57**	0.58**

**p < 0.01.

We explored the direct relationships between variables utilizing linear regression analysis. Results are presented in Table 3.

**Table 3:** Direct relationships between individual variable – linear regression.

Variables	*β*	Hypotheses
KC → QS	0.53**	H1 Supported
KC → QS (E-Qalin)	0.60**	H1a Supported
KC → QS (No E-Qalin)	0.47**	H1b Supported
KS → QS	0.37**	H2 Supported
KS → QS (E-Qalin)	0.28*	H2a Rejected
KS → QS (No E-Qalin)	0.38**	H2b Supported
KT → QS	0.53**	H3 Supported
KT → QS (E-Qalin)	0.44**	H3a Supported
KT → QS (No E-Qalin)	0.59**	H3b Supported
KI → QS	0.55**	H4 Supported
KI → QS (E-Qalin)	0.48**	H4a Supported
KI → QS (No E-Qalin)	0.61**	H4b Supported

*p < 0.05; **p < 0.01.

## 6. Discussion

This study reveals the interrelationship between teamwork, quality process, and KM. Teamwork aimed at continuous quality improvement and the acquisition of new knowledge by staff is the basis for understanding the quality of services in long-term care. With our research, we noticed an important difference that in E-Qalins’s quality management and KM, the work processes are based on multidisciplinary teamwork that is constantly taking place and always involves the residents’ representatives. Consequently, connections between colleagues are much better, as is the understanding of the work of other departments and a common focus on caring for the health, safety, and satisfaction of the residents (Kejžar et al., 2020).

We explored the direct relationship between knowledge creation and quality of services in NH. We found a significant and positive relationship between knowledge creation and quality of services, and quality of services with an E-Qalin certificate and quality of services without and E-Qalin certificate. Our results especially add value to managers in NH as we empirically supported that knowledge creation has a positive impact on the quality of services (Stojanović-Aleksić et al., 2019), which is also in line with previous research of McEvily and Chakravarthy (2000) and Tiwana (2004). Additionally, we explored the relationship between knowledge storage and quality of services in NH. We found a significant and positive relationship between knowledge storage and the quality of services in NH and quality of services without and E-Qalin certificate. There was no significant relationship between knowledge storage and the quality of services in NH with an E-Qalin certificate. With our results we partially extend the research of Alavi and Leidner (2001) that knowledge storage as an integral part of the overall KM process can positively influence outcomes in organization as is in our study elaborated as the quality of services that are offered in practice. We also aimed to establish whether knowledge transfer influences the quality of services in NH. The results showed a significant and positive correlation between knowledge transfer and quality of services, and quality of services with an E-Qalin certificate and quality of services without and E-Qalin certificate. Therefore, our empirical results offer support for the claims of Zaim et al. (2019) that argue that knowledge transfer enables better overall performance, which in our example is considered as the quality of services. Moreover, we argue that better knowledge transfer enables NH to have better quality of services, which is an extension of the research of Radević et al. (2021). To conclude our empirical research, we analyzed the relationship between knowledge implementation and the quality of services in NH. Our results displayed a significant and positive relationship between knowledge implementation and quality of services, and quality of services with an E-Qalin certificate and quality of services without and E-Qalin certificate. With our research we offer additional support to Mardani et al. (2018) that posit that knowledge implementation facilitates organizational performance, which is especially important in the context of NH as it influences the quality of services, which is in accordance with the research of Chan and Chao (2008).

During the research process, we found that all NH are aware of the importance of teamwork and knowledge in their institution, but do not approach to manage it strategically. In this area, the advantage of homes with an internal quality system is evident in the DEOS homes, where the quality manager for all homes takes care of organizational knowledge and real-time comparisons between expected and achieved indicators. Also, the results of our analysis show that the employees evaluate the knowledge storage in homes within the DEOS system better than in the E-Qalin quality management. The concepts themselves, such as management, storage of existing knowledge, and creation of new knowledge, are abstract to employees in the nursing department, since in practice they usually rely on the transfer of tacit knowledge while performing their work. The quality of services in NH is based on the achievement of standards in the nursing process, the transfer of tacit knowledge between staff, and the transfer of tacit knowledge into explicit knowledge. With explicit knowledge grows the organizational knowledge that enables the stable operation of the nursing process. Despite the standardized quality management in the E-Qalin quality system, the study pointed out the additional strategic importance of KM.

Another improvement opportunity seen in homes with their own quality system is close collaboration between homes (within the DEOS group). Homes that have E-Qalin certification could be linked in a similar way (in the form of regional links). During the COVID-19 period, collaboration between homes proved crucial, as homes within the DEOS group constantly collaborated and shared ideas and examples of best practices (including protective equipment), while others, mostly public homes, were left to their own ingenuity. In the article, we have emphasized the importance of involving the service users – the residents of the home – in planning their own care plan, and in making daily choices about how they want to spend their day. Only by involving residents can we implement personalized care as a new paradigm of service quality in NH (Stevens et al., 2019).

### 6.1. Theoretical implications

Our study has several implications to the literature on KM and quality management in NH. First, our results add to the body of literature that emphasizes the knowledge-based view of the organization (Kogut and Zander, 2003; Hislop and Helms, 2018) as we promote the crucial role of knowledge in the context of the functioning of NH. Second, we add to existing research that theoretically advances the aforementioned field with a conceptual model and empirically testing the phases of the KM process that can positively influence quality of services in NH (Grant, 1996; Martin and Javalgi, 2019). Third, with that we fill a gap, as the literature on KM in NH is scarce, and as we could see from the answers in our study so is the knowledge about KM in NH. Moreover, this paper shows that tacit knowledge does have its place in NH practice, but it needs to be supported by a wider workplace learning framework to maximize the potential for consistent good care in NH. Greater awareness about the importance of tacit knowledge in relation to nursing practice as explored in this study may contribute to the acknowledgement that teamwork increases transfer of knowledge and benefits toward creating a stable working environment and to daily changes and adaptations in care processes. With teamwork, we especially raise awareness about set goals and encourage creative and innovative solutions in the daily implementation of services. Our findings are in line with Erhardt (2011) that teamwork is always good and has the potential to achieve, maintain, and even improve the quality of services in NH.

### 6.2. Practical implications

Practical implications are aimed at managers, directors, and practitioners in the environment of NH. The findings of our research present opportunities for improvements in NH, through better understanding and knowledge of KM, quality management, and teamwork as factors that influence the quality of services provided to users in practice. Based on empirical findings, our study helps stakeholders in understanding the importance of KM activities and teamwork to encourage knowledge creation, knowledge storage, knowledge transfer, and knowledge implementation in the ever-changing environment of NH. Problems that can seriously threaten the quality of care services in NH are related to lack of well-trained nursing staff, frequent new jobs, and the employment of foreign workers, where the role of managers is especially essential. Moreover, the role of teamwork here is key in promoting the transfer of expertise, which in turn involves knowledge of the residents and their care plan. In a similar vein, managers in NH have to also understand why the authocratic system of management is not well aligned to encourage knowledge transfer, and what to do with all of the ideas that arise at team meetings in organizations. Team members’ perception during day-to-day teamwork through the lens of complexity science helps us to understand how and why caregivers behave in this way as a “perceptual information guides our decision and actions, and shapes our beliefs” (Pype et al., 2018). Teamwork is therefore the basis of work in nursing homes, enabling knowledge management, the transmission of values and the promotion of the creation of new knowledge, thus constantly improving the quality of services. Factors such as the shortage of well-trained caregivers, the high turnover of staff and the role of the user, who becomes a co-designer of his own care plan, require that management be prepared for a strategic approach to knowledge management.

Another important practical implication is focused on the knowledge of users of services – residents – which can lead to quality services that comply with care protocols on one hand and are tailored to the user on the other. Residents are the ones we can learn from – their wishes, expectations, needs, and their own perceptions of quality in aging in NH. With the awareness that users are the ones who determine the quality of services, we are shifting the paradigm of determining quality from the perspective of experts to a co-creative social model in which we research together with users. This is why the teamwork method, where we also involve residents in finding better solutions, is so important. By involving residents and taking their opinions into account, we move from the role of an expert planning the process of care and support within the system to the role of a partner in the process of planning everyday life in the NH. As residents’ expectations, desires and goals change over the generations, it is also necessary to change the culture of the NH – from planning the process of service delivery for residents to planning with residents.

### 6.3. Limitations and future research directions

Even with the theoretical and practical implications of our research, we need to address limitations of our study as well. First, in our study, we included four homes, which hinders the generalizability of our findings. To overcome this limitation, we propose to conduct similar research on a bigger sample size of more than 100 NH in Slovenia, or to also extend it to foreign countries in order to increase the reliability and validity of our research findings. Second, one limitation is that there is lack of awareness, understanding, and knowledge with the concept of KM in NH, both from managers and nursing staff. One potential solution is to develop common terminology to reduce potential bias in answers provided by respondents. Third, the methodological issue of common method bias should be taken into account, which can be understood as a limitation of our model. In a similar vein, researching only direct relationships between dependent and independent model can be viewed as a limitation of our study, which can be overcome by exploring potential moderating effects of different variables on the hypothesized relationships in our model. Furthermore, we believe that measuring the concepts of quality management and teamwork would add additional value to our hypothesized research model. Fourth, the complexity of the environment of NH potentially hinders the responses from our respondents as they are continuously working in difficult conditions and in a complex and demanding profession.

For future research, we recommend follow-up studies and more prior training on the topic as it would increase building a KM ecosystem in NH in a systematic and constant manner. With organizational elements such as formal and informal communication, regular teamwork (with residents), information systems, written protocols, a personalized care plan for residents, NH becomes a system of knowledge in long-term care. In addition, future research might explore potential moderating effects of organizational factors such as leadership style and information-communication technologies on the relationship between KM and quality of services in NH. For future research, it might be beneficial to distinguish between the differences in NH, such as organizational size measured by the number of employees, which can be understood also as an additional control variable in the study. Considering the complexity of the NH and its profession, it could be beneficial to apply also a qualitative research design such as in-depth semi-structured interviews or case studies to gain more in-depth insight into specific research constructs that were utilized in our study. Moreover, in order to overcome the limitation of the study that is conducted only in one country, we suggest to conduct a follow-up study on an international sample.

## 7. Conclusion

This paper has addressed the impact of KM in NH in relationship with a quality management system where teamwork plays a crucial role in the process of continuous development and implementing services in NH. To provide a high quality of living to the residents, well-trained staff equipped with relevant knowledge are critical in performing the various caring activities in NH. Teamwork is considered as a knowledge-integrated mechanism to increase knowledge transfer, storage, implementation of existing knowledge, and creation of new knowledge. Person centered care and continuous quality improvement in NH, means a higher quality of life for residents. Nursing homes have to adapt to changes in society, but their most important focus is on the personalisation of care processes to its residents. So far, several reports have demonstrated how NH utilized the knowledge of residents’ “personhood” to personalize their care and to enhance their well-being (Ettelt et al., 2020). Therefore, we believe that involving residents in planning an individualized care and living plan is essential to improving the quality of care and support services. Having a core team of committed, reliable, long-term nursing staff who share their knowledge and NH values is the mission for NH management. The presented quality management structure helps to transfer the knowledge, tacit and explicit, on an organizational level with teamwork and encourages innovation processes in NH. Nursing homes management needs more knowledge about teamwork and the impact of teamwork on KM in NH.

To conclude, the implementation of KM in NH, with an emphasis on the teamwork that encourages the transfer of knowledge and creation of new knowledge, has the potential to empower caregivers to work not just by their instinct, but by values, protocols, and individualized care plans. The NH must feel like a family home and provide quality, person-centered care. It emphasizes the need for expertise in care, but also for compassion and empathy (Stevens et al., 2019). All of this leads to improved resident well-being, higher quality of care, and greater storage of organizational knowledge. In times of frequent job changes of caregivers and nurses in NH, and shortages of appropriately qualified nursing staff, it is crucial to provide an ecosystem for the creation of organizational knowledge and to introduce teamwork among caregivers as “that’s how we work here.” By incorporating the knowledge of staff and ideas of residents, we create a creative work and homelike environment in NH. “KM and quality management is rarely explored within the context of NH, thus our results extend the discussion regarding the aforementioned concepts within the NH environment, which represents the added value of our research and a unique contribution to the existing body of literature that elaborates on the influence of KM and quality management on quality of services within NH.”

## Data availability statement

The datasets presented in this study can be found in online repositories. The names of the repository/repositories and accession number(s) can be found at: https://doi.org/10.6084/m9.figshare.21814596.

## Author contributions

AK, VD, and SC conceptualized and designed the study. AK gathered the data. VD and SC analyzed the data. All authors contributed to the article and approved the submitted version.

## Funding

VD and SC acknowledge the funding by the Slovenian Research Agency programme SEB ULP5-0364 (A). AK acknowledges funding by the Erasmus + Capacity Building in Higher Education 2020 nEUROcare project, ref. 618596-EPP-1-2020-1-SE-EPPKA2-CBHE-JP.

## Conflict of interest

The authors declare that the research was conducted in the absence of any commercial or financial relationships that could be construed as a potential conflict of interest.

## Publisher’s note

All claims expressed in this article are solely those of the authors and do not necessarily represent those of their affiliated organizations, or those of the publisher, the editors and the reviewers. Any product that may be evaluated in this article, or claim that may be made by its manufacturer, is not guaranteed or endorsed by the publisher.

## References

[ref1] Abdullah A. Date H. (2009). Public sector knowledge management: a generic framework. Publ. Sect. ICT Manag. Rev. 3, 1–14.

[ref2] Abou Hashish E. A. (2017). Research and knowledge transfer. Bus. Econ. J. 8:e109.

[ref3] Akoglu H. (2018). User’s guide to correlation coefficients. Turkish J. Emerg. Med. 18, 91–93. doi: 10.1016/j.tjem.2018.08.001, PMID: 30191186 PMC6107969

[ref4] Al Ahbabi S. A. Singh S. K. Balasubramanian S. Gaur S. S. (2018). Employee perception of impact of knowledge management processes on public sector performance. J. Knowl. Manag. 23, 351–373. doi: 10.1108/JKM-08-2017-0348

[ref5] Alavi M. Leidner D. E. (2001). Review: knowledge management and knowledge management systems: conceptual foundations and research issues. MIS Q. 25, 107–136. doi: 10.2307/3250961

[ref6] Alegre J. Sengupta K. Lapiedra R. (2013). Knowledge management and innovation performance in a high-tech SMEs industry. Int. Small Bus. J. 31, 454–470. doi: 10.1177/0266242611417472

[ref7] Anand A. Muskat B. Creed A. Zutshi A. Csepregi A. (2021). Knowledge sharing, knowledge transfer and SMEs: evolution, antecedents, outcomes and directions. Pers. Rev. 50, 1873–1893. doi: 10.1108/PR-05-2020-0372

[ref8] André B. Grønning K. Jacobsen F. F. Haugan G. (2021). “Joy of life” in nursing homes. Healthcare personnel experiences of the implementation of the national strategy. A qualitative study with content analysis of interviews. BMC Health Serv. Res. 21:771. doi: 10.1186/s12913-021-06801-w 34348715 PMC8335868

[ref9] Argote L. Beckman S. L. Epple D. (1990). The persistence and transfer of learning in industrial settings. Manag. Sci. 36, 140–154. doi: 10.1287/mnsc.36.2.140

[ref10] Argote L. Fahrenkopf E. (2016). Knowledge transfer in organizations: the roles of members, tasks, tools, and networks. Organ. Behav. Hum. Decis. Process. 136, 146–159. doi: 10.1016/j.obhdp.2016.08.003

[ref11] Argote L. Miron-Spektor E. (2011). Organizational learning: from experience to knowledge. Organ. Sci. 22, 1123–1137. doi: 10.1287/orsc.1100.0621

[ref12] Bernerth J. B. Aguinis H. (2016). A critical review and best-practice recommendations for control variable usage. Pers. Psychol. 69, 229–283. doi: 10.1111/peps.12103

[ref13] Bolisani E. Bratianu C. (2017). Knowledge strategy planning: an integrated approach to manage uncertainty, turbulence, and dynamics. J. Knowl. Manag. 21, 233–253. doi: 10.1108/JKM-02-2016-0071

[ref14] Bosch-Sijtsema P. M. Ruohomäki V. Vartiainen M. (2009). Knowledge work productivity in distributed teams. J. Knowl. Manag. 13, 533–546. doi: 10.1108/13673270910997178

[ref15] Butler T. Feller J. Pope A. Emerson B. Murphy C. (2008). Designing a core IT artefact for knowledge management systems using participatory action research in a government and a non-government organization. J. Strateg. Inf. Syst. 17, 249–267. doi: 10.1016/j.jsis.2007.10.002

[ref16] Cammer A. Morgan D. Stewart N. McGilton K. Rycroft-Malone J. Dopson S.. (2014). The hidden complexity of long-term care: how context mediates knowledge translation and use of best practices. Gerontologist 54, 1013–1023. doi: 10.1093/geront/gnt068, PMID: 23856027

[ref17] Castle N. G. Ferguson J. C. (2010). What is nursing home quality and how is it measured? Gerontologist 50, 426–442. doi: 10.1093/geront/gnq052, PMID: 20631035 PMC2915498

[ref18] Chan I. Chao C. K. (2008). Knowledge management in small and medium-sized enterprises. Commun. ACM 51, 83–88. doi: 10.1145/1330311.1330328

[ref19] Chen C. J. Huang J. W. (2009). Strategic human resource practices and innovation performance–the mediating role of knowledge management capacity. J. Bus. Res. 62, 104–114. doi: 10.1016/j.jbusres.2007.11.016

[ref20] Chen S. H. Tao C. Q. He W. (2012). Empirical research on relationship of knowledge integration and innovation ability of IT enterprise. Int. J. Netw. Virtual Org. 11, 315–328. doi: 10.1504/IJNVO.2012.048913

[ref21] Choi B. Lee H. (2002). Knowledge management strategy and its link to the knowledge creation process. Expert Syst. Appl. 23, 173–187. doi: 10.1016/S0957-4174(02)00038-6

[ref22] Dawson R. (2001). Knowledge capabilities as the focus of organizational development and strategy. J. Knowl. Manag. 4, 320–327. doi: 10.1108/13673270010379876

[ref23] DEOS (2022). Integrated care for elderly. Available at: https://www.deos.si/ (Accessed October 3, 2022).

[ref24] Donate M. Pablo J. S. (2015). The role of knowledge-oriented leadership in knowledge management practices and innovation. J. Bus. Res. 68, 360–370. doi: 10.1016/j.jbusres.2014.06.022

[ref25] Du J. Lin X. Cai Y. Sun F. Amankwah-Amoah J. (2021). When teamwork works: examining the relationship between leader-member exchange differentiation and team creativity. Front. Psychol. 12:646514. doi: 10.3389/fpsyg.2021.646514 35126217 PMC8815316

[ref26] Erhardt N. (2011). Is it all about teamwork? Understanding processes in team-based knowledge work. Manag. Learn. 42, 87–112. doi: 10.1177/1350507610382490

[ref27] Ettelt S. Damant J. Perkins M. Raphael W. L. W. (2020). Personalisation in Care Homes For Older People Final report. London: Policy Innovation and Evaluation Research Unit (PIRU).

[ref28] Filipovič Hrast M. Rakar T. (2021). Care policy in Slovenia: divergent trends and convergent attitudes. Revija za socijalnu politiku 27, 303–321. doi: 10.3935/rsp.v28i3.1802

[ref29] Firis (2022). About E-Qalin. Available at: http://www.firis-imperl.si/izobrazevanje/e-qalin/#e-qalin-gmbh-evropsko-zdruzenje-e-qalin (Accessed October 3, 2022).

[ref30] Gabriel M. Stanley I. Saunders T. (2017). Open Innovation in Health. Available at: http://allcatsrgrey.org.uk/wp/download/management/organisational_development/open_innovation_in_health.pdf (Accessed October 3, 2022).

[ref31] Gaviria-Marin M. Merigó J. M. Baier-Fuentes H. (2019). Knowledge management: A global examination based on bibliometric analysis. Technol. Forecast. Soc. Change 140, 194–220. doi: 10.1016/j.techfore.2018.07.006

[ref32] Girard J. Girard J. (2015). Defining knowledge management: toward an applied compendium. Online J. Appl. Knowl. Manag. 3, 1–20.

[ref33] Gold A. H. Malhotra A. Segars A. H. (2001). Knowledge management: an organizational capabilities perspective. J. Manag. Inf. Syst. 18, 185–214. doi: 10.1080/07421222.2001.11045669

[ref34] Gorry G. A. (2008). Sharing knowledge in the public sector: two case studies. Knowl. Manag. Res. Pract. 6, 105–111. doi: 10.1057/palgrave.kmrp.8500172

[ref35] Grant R. M. (1996). Towards a knowledge-based view of the firm. Strateg. Manag. J. 17, 109–122. doi: 10.1002/smj.4250171110

[ref36] Grødal K. Innstrand S. T. Haugan G. André B. (2019). Affective organizational commitment among nursing home employees: a longitudinal study on the influence of a health-promoting work environment. Nurs. Open 6, 1414–1423. doi: 10.1002/nop2.338, PMID: 31660169 PMC6805324

[ref37] Hall E. O. Høy B. (2012). Re-establishing dignity: nurses’ experiences of caring for older hospital patients. Scand. J. Caring Sci. 26, 287–294. doi: 10.1111/j.1471-6712.2011.00931.x, PMID: 22011324

[ref38] Hansen M. T. (1999). The search-transfer problem: the role of weak ties in sharing knowledge across organization subunits. Admin. Sci. Quart. 44, 82–111. doi: 10.2307/2667032

[ref39] Harman H. H. (1976). Modern Factor Analysis. 3rd Edn. Chicago: University of Chicago Press.

[ref40] Hassan N. A. H. M. Noor M. N. M. Hussin N. (2017). Knowledge transfer practice in organization. Int. J. Acad. Res. Bus. Soc. Sci. 7, 750–762. doi: 10.6007/IJARBSS/v7-i8/3291

[ref41] Hislop D. R. B. Helms R. (2018). Knowledge Management in Organizations: A Critical Introduction. Oxford: Oxford University Press.

[ref42] Hlebec V. Rakar T. (2017). “Aging policies in Slovenia: before and after austerity” in Selected Contemporary Challenges of Ageing Policy. eds. Tomczyk Ł. Klimczuk A. (Kraków: Uniwersytet Pedagogiczny w Krakowie), 27–52.

[ref43] Ibidunni A. S. Atolagbe T. M. Obi J. Olokundun M. A. Oke O. A. Amaihian A. B.. (2018). Moderating effect of entrepreneurial orientation on entrepreneurial competencies and performance of agro-based SMEs. Int. J. Enterpren. 22, 1–9.

[ref44] Joshi K. D. Sarker S. Sarker S. (2007). Knowledge transfer within information systems development teams: examining the role of knowledge source attributes. Decis. Support. Syst. 43, 322–335. doi: 10.1016/j.dss.2006.10.003

[ref45] Kejžar A. Filipovič Hras M. Mali J. Škrlj M. Šabec E. Malek N. (2020). Prenos znanja v socialnem zavodu; raziskovalno poročilo. Ljubljana: Fakulteta za socialno delo.

[ref46] Kejžar A. Rihter L. Sajovic J. Drevenšek G. (2022). Nutrition and congruent care improve wellbeing of residents with dementia in Slovenian care homes. Front. Nutr. 9:796031. doi: 10.3389/fnut.2022.796031 35308276 PMC8931699

[ref47] Kogut B. Zander U. (2003). Knowledge of the firm and the evolutionary theory of the multinational corporation. J. Int. Bus. Stud. 34, 516–529. doi: 10.1057/palgrave.jibs.8400058

[ref48] Konno N. Schillaci C. E. (2021). Intellectual capital in society 5.0 by the lens of the knowledge creation theory. J. Intellect. Cap. 22, 478–505. doi: 10.1108/JIC-02-2020-0060

[ref49] Kumar J. Ganesh L. (2009). Research on knowledge transfer in organizations: a morphology. J. Knowl. Manag. 13, 161–174. doi: 10.1108/13673270910971905

[ref50] Lee V. H. Leong L. Y. Hew T. S. Ooi K. B. (2013). Knowledge management: a key determinant in advancing technological innovation? J. Knowl. Manag. 17, 848–872. doi: 10.1108/JKM-08-2013-0315

[ref51] Li M. Liu H. Zhou J. (2018). G-SECI model-based knowledge creation for CoPS innovation: the role of Grey knowledge. J. Knowl. Manag. 22, 887–911. doi: 10.1108/JKM-10-2016-0458

[ref52] Mahmoudsalehi H. Moradkhannejad R. Safari K. (2012). How knowledge management is affected by organizational structure. Learn. Organ. 19, 518–528. doi: 10.1108/09696471211266974

[ref53] Mardani A. Nikoosokhan S. Moradi M. Doustar M. (2018). The relationship between knowledge management and innovation performance. J. High Technol. Managem. Res. 29, 12–26. doi: 10.1016/j.hitech.2018.04.002

[ref54] Martelo-Landroguez S. Cepeda-Carrión G. (2016). How knowledge management processes can create and capture value for firms? Knowl. Manag. Res. Pract. 14, 423–433. doi: 10.1057/kmrp.2015.26

[ref55] Martin S. L. Javalgi R. G. (2019). Explaining performance determinants: a knowledge-based view of international new ventures. J. Bus. Res. 101, 615–626. doi: 10.1016/j.jbusres.2019.02.041

[ref56] McEvily S. Chakravarthy B. (2000). The persistence of knowledge-based advantage: an empirical test for product performance and technological knowledge. Strateg. Manag. J. 23, 285–305. doi: 10.1002/smj.223

[ref57] McEvoy P. J. Ragab M. A. F. Arisha A. (2019). The effectiveness of knowledge Management in the public sector. Knowl. Manag. Res. Pract. 17, 39–51. doi: 10.1080/14778238.2018.1538670

[ref58] Mitchell R. Boyle B. (2010). Knowledge creation measurement methods. J. Knowl. Manag. 14, 67–82. doi: 10.1108/13673271011015570

[ref59] Obeidat B. Y. Al-Suradi M. M. Masa’deh R. Tarhini A. (2016). The impact of knowledge management on innovation: an empirical study on Jordanian consultancy firms. Manag. Res. Rev. 39, 1214–1238. doi: 10.1108/MRR-09-2015-0214

[ref60] Oyemomi O. Liu S. Neaga I. Alkhuraiji A. (2016). How knowledge sharing and business process contribute to organizational performance: using the fs QCA approach. J. Bus. Res. 69, 5222–5227. doi: 10.1016/j.jbusres.2016.04.116

[ref61] Parker R. Bradley L. (2000). Organisational culture in the public sector: evidence from six Organisations. Int. J. Public Sect. Manag. 13, 125–141. doi: 10.1108/09513550010338773

[ref62] Phelan A. McCormack B. (2016). Exploring nursing expertise in residential care for older people: a mixed method study. J. Adv. Nurs. 72, 2524–2535. doi: 10.1111/jan.13001, PMID: 27174409

[ref63] Podsakoff P. M. Bommer W. H. Podsakoff N. P. MacKenzie S. B. (2006). Relationships between leader reward and punishment behavior and subordinate attitudes, perceptions, and behaviors: a meta-analytic review of existing and new research. Organ. Behav. Hum. Decis. Process. 99, 113–142. doi: 10.1016/j.obhdp.2005.09.002

[ref64] Purkis M. E. Bjornsdottir K. (2006). Intelligent nursing: accounting for knowledge as action in practice. Nurs. Philos. 7, 247–256. doi: 10.1111/j.1466-769X.2006.00283.x, PMID: 16965306

[ref65] Pype P. Mertens F. Helewaut F. Krystallidou D. (2018). Healthcare teams as complex adaptive systems: understanding team behaviour through team members’ perception of interpersonal interaction. BMC Health Serv. Res. 18, 1–13. doi: 10.1186/s12913-018-3392-3 30029638 PMC6053823

[ref66] Rabeea O. M. Nassar I. A. Almsafir M. K. (2019). Knowledge management processes and sustainable competitive advantage: an empirical examination in private universities. J. Bus. Res. 94, 320–334. doi: 10.1016/j.jbusres.2018.02.013

[ref67] Radević I. Dimovski V. Lojpur A. Colnar S. (2021). Quality of healthcare services in focus: the role of knowledge transfer, hierarchical organizational structure and trust. Knowl. Manag. Res. Pract., 1–12. doi: 10.1080/14778238.2021.1932623

[ref68] Reagans R. Argote L. Brooks D. (2005). Individual experience and experience working together: predicting learning rates from knowing who knows what and knowing how to work together. Manag. Sci. 51, 869–881. doi: 10.1287/mnsc.1050.0366

[ref69] Rinnan E. André B. Drageset J. Garåsen H. Espnes G. A. Haugan G. (2018). Joy of life in nursing homes: a qualitative study of what constitutes the essence of joy of life in elderly individuals living in Norwegian nursing homes. Scand. J. Caring Sci. 32, 1468–1476. doi: 10.1111/scs.12598, PMID: 30070384

[ref70] Samoilenko N. Nahar N. (2013). “IT tools for knowledge storage and retrieval in globally distributed complex software and systems development of high-tech organizations,” in *2013 Proceedings of PICMET ‘13: Technology Management in The IT-driven Services (PICMET)*. 1353–1369.

[ref71] Sandvoll A. M. (2017). Tacit practice in care homes. Int. Pract. Dev. J. 7, 1–13. doi: 10.19043/ipdj.7SP.006

[ref72] Senior T. J. (2019). Open to all: dementia, creativity, and open ecosystem innovation. Front. Sociol. 4:10. doi: 10.3389/fsoc.2019.00010, PMID: 33869337 PMC8022449

[ref73] Sheng M. L. Chang S. Teo T. Lin Y. (2013). Knowledge barriers, knowledge transfer, and innovation competitive advantage in healthcare settings. Manag. Decis. 51, 461–478. doi: 10.1108/00251741311309607

[ref74] Stein E. W. Zwass V. (1995). Actualizing organizational memory with information systems. Inf. Syst. Res. 6, 85–117. doi: 10.1287/isre.6.2.85

[ref75] Stevens M. Moriarty J. Harris J. Hussein S. Cornes M. (2019). Social care managers and care workers’ understanding of personalisation in older people’s services. Work. Older People 23, 37–45. doi: 10.1108/WWOP-11-2018-0022

[ref76] Stojanović-Aleksić V. Erić Nielsen J. Bošković A. (2019). Organizational prerequisites for knowledge creation and sharing: empirical evidence from Serbia. J. Knowl. Manag. 23, 1543–1565. doi: 10.1108/JKM-05-2018-0286

[ref77] Szulanski G. (1996). Exploring internal stickiness: impediments to the transfer of best practice within the firm. Strat. Manag. J. 17, 27–43. doi: 10.1002/smj.4250171105

[ref78] Taraszewski S. A. (2017). Understanding Knowledge Storage/Retrieval System Success: An Analytic Network Process Perspective. Doctoral dissertation. Cleveland: Cleveland State University OhioLINK Electronic Theses and Dissertations Center.

[ref79] Tassabehji R. Mishra J. L. Dominguez-Perry C. (2019). Knowledge sharing for innovation performance improvement in micro/SMEs: an insight from the creative sector. Prod. Plann. Contr. 30, 935–950. doi: 10.1080/09537287.2019.1582101

[ref80] Tirrito T. (2003). Aging in the New Millennium: A Global View. South Carolina: University of South Carolina Press.

[ref81] Tiwana A. (2004). An empirical study of the effect of knowledge integration on software development performance. Inform. Softw. Technol. 46, 899–906. doi: 10.1016/j.infsof.2004.03.006

[ref82] Tiwari N. Chilwal A. (2014). On minimum variance optimal controlled sampling: A simplified approach. J. Stat. Theor. Pract. 8, 692–706. doi: 10.1080/15598608.2013.828338

[ref83] Toppl L. Barker B. Degenhardt L. (2004). The external validity of results derived from ecstasy users recruited using purposive sampling strategies. Drug Alcohol Depend. 73, 33–40. doi: 10.1016/j.drugalcdep.2003.09.001, PMID: 14687957

[ref84] Tourangeau A. E. Patterson E. Saari M. Thomson H. Cranley L. (2017). Work-related factors influencing home care nurse intent to remain employed. Health Care Manag. Rev. 42, 87–97. doi: 10.1097/HMR.0000000000000093, PMID: 26545207

[ref85] Van den Hooff B. De Ridder J. A. (2004). Knowledge sharing in context: the influence of organizational commitment, communication climate and CMC use on knowledge sharing. J. Knowl. Manag. 8, 117–130. doi: 10.1108/13673270410567675

[ref86] Wong Y. K. Aspinwall E. (2005). An empirical study of the important factors for knowledge-management adoption in the SME sector. J. Knowl. Manag. 9, 64–82. doi: 10.1108/13673270510602773

[ref87] Xuan V. N. (2020). Factors affecting knowledge sharing in enterprises: evidence from small and medium Enterprises in Vietnam. Manag. Sci. Lett. 10, 469–478. doi: 10.5267/j.msl.2019.8.023

[ref88] Zaim H. Muhammed S. Terim M. (2019). Relationship between knowledge management processes and performance: critical role of knowledge utilization in organizations. Knowl. Manag. Res. Pract. 17, 24–38. doi: 10.1080/14778238.2018.1538669

